# How does career calling influence doctoral students' academic resilience:a moderated serial mediation model

**DOI:** 10.3389/fpsyg.2025.1621479

**Published:** 2026-01-14

**Authors:** Chibin Zhang, Dexian Li

**Affiliations:** Faculty of Education, Liaoning Normal University, Dalian, China

**Keywords:** academic resilience, career adaptability, career calling, decent work, doctoral students

## Abstract

Academic resilience, as a core competency reflecting perseverance and positive adaptation in academic adversity, demonstrates doctoral students' enduring passion and vitality for academic careers. Grounded in Career Construction Theory and Ecological Systems Theory, this study aims to empirically test a theoretically derived moderated serial mediation model explaining how and under what conditions career calling fosters academic resilience among doctoral students. Data were collected from 865 doctoral students using the Career Calling Scale, Career Adapt-Abilities Scale–Short Form, Perceived Future Decent Work Scale, Academic Resilience Scale, and Decent Work Scale. Results showed that career calling was positively associated with academic resilience; career adaptability partially mediated the relationship between career calling and academic resilience; perceived future decent work partially mediated the relationship between career calling and academic resilience; career adaptability and perceived future decent work exerted a serial mediating effect in the impact of career calling on academic resilience; decent work positively moderated the relationships between career calling and academic resilience, career adaptability and academic resilience, and perceived future decent work and academic resilience. These findings extend theoretical understanding by integrating individual and contextual perspectives to reveal the multi-layered mechanisms of resilience formation in doctoral education, while offering actionable implications for enhancing calling education, fostering adaptability, and improving decent-work conditions. However, as the study relied on cross-sectional self-report data from Chinese doctoral students, future research should employ longitudinal and cross-cultural designs to strengthen causal inference and generalizability.

## 1 Introduction

In recent years, doctoral education has faced intensifying structural pressures that heighten uncertainty for early-career scholars, such as institutional bureaucratization, the commodification of knowledge production, escalating performance regimes, and the expansion of PhD cohorts without commensurate growth in tenure-track positions, which together render academic career paths increasingly precarious, particularly for young researchers navigating the transition from training to independent scholarship. These dynamics aggravate the risks of attrition and psychological strain during training. Empirical work shows that a substantial share of doctoral candidates discontinue for academic reasons—difficulty integrating into programs, supervisory breakdowns, loss of interest, or misalignment of plans (Lovitts, 2002)—and many report emotional exhaustion or depressive symptoms (McCray and Joseph-Richard, 2020). Such patterns underscore the need to understand and strengthen personal resources that help doctoral students sustain progress under prolonged uncertainty.

Academic resilience, an extension and evolution of psychological resilience in the educational domain, traces its conceptual roots to early formulations of resilience that emphasized adaptive responses to stress and adversity. Martin (2013) later expanded this to academia, defining it as the ability of students or researchers to achieve academic success by modifying rules, operations, or plans when confronted with adversities such as academic pressures and challenges. Resilience has been documented as an essential component in managing stress (Ang et al., 2021). Given the protracted and uncertain nature of academic careers, doctoral students must endure rigorous training, arduous research processes (Chamadia and Qureshi, 2021), and stringent thesis writing and evaluation procedures. Throughout this journey, academic resilience becomes a pivotal force sustaining their perseverance (González, 2007). It enables doctoral students to maintain enthusiasm and conviction in their research goals despite setbacks, adapt strategies, seek external support, and self-motivate, ultimately overcoming obstacles to attain academic accomplishments.

Career calling is defined as “a transcendent summons experienced beyond self-interest, actualized through a sense of purpose or meaning, and oriented toward prosocial values and goals as primary motivators” (Dik and Duffy, 2009). Individuals with a career calling perceive their work and life as an integrated whole, characterized by profound responsibility and meaningfulness that transcends mere livelihood. Such individuals view work as a means to realize personal values and societal contributions. Empirical studies demonstrate that career calling positively impacts psychological and behavioral outcomes, enhancing work engagement, performance (Zhang et al., 2015), life satisfaction, and psychological resilience, while reducing burnout (Wen et al., 2024). However, existing research on career calling and resilience lacks focus on doctoral students, leaving its influence on academic resilience underexplored.

Building on this analysis, this study focuses on doctoral students and constructs a moderated chain mediation model grounded in Career Construction Theory and Ecological Systems Theory. The model aims to elucidate the mechanisms through which career calling influences academic resilience, thereby advancing theoretical understanding and practical applications of academic resilience in higher education contexts and providing robust theoretical and practical guidance for fostering doctoral students' holistic development and scholarly achievements.

## 2 Literature review and hypotheses

### 2.1 The relationship between career calling and academic resilience

Career calling refers to an individual's transcendent perception of their professional and life pursuits, with altruistic values serving as the primary motivational source, aiming to integrate personal career choices with life meaning and goals to fulfill one's role (Duffy et al., 2018). Generally, individuals with career calling view work as an integral part of their lives. Their dedication stems not from external rewards but from the pursuit of life purpose and meaning. Such individuals demonstrate clear goals, strong passion, and heightened responsibility toward their mission-driven domains (Duffy et al., 2011), which enhances positive cognitive and emotional evaluations of work (Dik and Duffy, 2009). For specific populations, research shows that college students' career calling positively predicts work meaning, academic satisfaction (Duffy et al., 2014), life satisfaction (Duffy and Sedlacek, 2010), among working adults, calling has also been shown to predict higher career satisfaction (Xie et al., 2016). Further research indicates that individuals with a calling derive greater psychological capital (hope, self-efficacy, resilience, and optimism) from their work experiences (Choi et al., 2018). More direct evidence reveals that occupational calling positively predicts occupational resilience among nurses (Xie et al., 2024). A study of medical students found that even those with lower self-efficacy can still demonstrate high professional commitment if they possess strong occupational calling (Goodin et al., 2014). Additionally, sense of purpose—a core component of career calling - serves as a critical factor in fostering doctoral students' resilience, enabling them to persevere through academic challenges and achieve breakthroughs (Chamadia and Qureshi, 2021).

Therefore, this study proposes Hypothesis 1 (H1): career calling positively associated with academic resilience.

### 2.2 The mediating role of career adaptability

As a core component of career construction theory (Savickas, 2005), career adaptability represents an individual's essential capacity to respond to external challenges during career development. It reflects one's preparedness to adjust to various career tasks and role transitions, facilitating successful career transitions and professional achievements (Haenggli and Hirschi, 2020; Ramos and Lopez, 2018). Specifically, it denotes the dynamic ability to address current and future career demands, including the flexibility to modify career goals, skills, and attitudes in response to changing professional environments (Savickas and Porfeli, 2012). Career adaptability is considered a career adaptation resource influenced by both individual traits and external environmental factors. Career adaptation behaviors and outcomes emerge from the interaction between career adaptability and career adaptation preparedness, shaped through dimensions of concern, control, curiosity, and confidence (Savickas, 2005; Savickas and Porfeli, 2012). Regarding the relationship between career calling and career adaptability, studies on older adults, and college students have consistently demonstrated positive correlations (Chen and Zhang, 2023). As a crucial psychosocial resource for career development, career adaptability constitutes an individual's “response readiness” to both predictable and unpredictable career situations (Savickas, 1997), inherently reflecting proactive coping capabilities. Research on employees of higher-education institutes demonstrates a direct positive correlation between career adaptability and career resilience (Ahmad et al., 2023). Similarly, studies focusing on nursing interns have also identified a direct association between these two factors (Xu Y., et al., 2024).

Thus, Hypothesis 2 (H2): career adaptability positively mediates the relationship between career calling and academic resilience.

### 2.3 The mediating role of perceived future decent work

Decent work refers to productive employment opportunities obtained under conditions of freedom, equity, safety, and human dignity, with its core being the promotion of workplace rights, employment equality, social protection, and social dialogue (International Labour Organization, 1999). Recent studies rooted in the Psychology of Working Theory (PWT) have highlighted decent work as a multifaceted construct, integrating dimensions such as physical safety, fair remuneration, access to healthcare, reasonable working hours, and organizational values aligned with family and society (Duffy et al., 2016, 2017). Perceived future decent work builds upon the concept of decent work (Wei et al., 2022), referring to workers' perceptions of future work environments. According to PWT, antecedents of perceived future decent work may include marginalization, economic constraints (Duffy et al., 2016). Research has found that work volition among college students from impoverished families directly predicts perceptions of future decent work (Wei et al., 2022).

Regarding outcome variables, decent work can directly or indirectly influence wellbeing by fulfilling basic human needs. Moreover, perceptions of future decent work demonstrate similar effects, particularly in mediating pathways between motivational factors like career calling and adaptive outcomes such as academic resilience. Studies focusing on pre-service college students (those yet to enter the workforce) have shown that future decent work perceptions are positively correlated with academic satisfaction (Wen et al., 2023) and negatively correlated with employment anxiety (Wan et al., 2024), suggesting their role in buffering stressors and enhancing persistence in academic pursuits. Further research indicates that these perceptions also serve as a significant positive predictor of resilience (Wen et al., 2024), implying that optimistic views of future work can foster the psychological resources needed to overcome academic challenges, thereby supporting their potential mediating function in linking career calling—a sense of purpose in one's vocational path—to greater resilience. Complementary evidence from Nigerian youth research shows that work volition (a construct related to career calling) predicts future decent work opportunities, and those with better decent work prospects exhibit higher academic satisfaction and career engagement (Ezema and Autin, 2024)—implying that career-related aspirations may translate to academic persistence via enhanced future labor expectations. Moreover, the future dimension of decent work shares isomorphism with hope, as both center on positive future expectations. Given that hope correlates with reduced distress and enhanced adaptive functioning (Feldman and Snyder, 2005; Arnau et al., 2007), this parallel suggests perceived future decent work could mediate the positive effect of career calling on academic resilience by fostering adaptive psychological resources.

Accordingly, Hypothesis 3 (H3): perceived future decent work positively mediates the relationship between career calling and academic resilience.

### 2.4 The chain mediation effect of career adaptability and perceived future decent work

Career adaptability, as a central construct in Career Construction Theory (Savickas, 2005), represents individuals' psychosocial resources for managing career transitions and constructing meaningful vocational paths through ongoing interactions between their internal self-concepts and external environments. According to CCT, career adaptability facilitates the integration of personal values, skills, and environmental opportunities, enabling individuals to form coherent narratives about their future careers, including optimistic perceptions of securing decent work. This theoretical perspective posits that adaptable individuals are better equipped to anticipate and prepare for future work conditions, thereby enhancing their expectations of achieving decent work characterized by fair pay, safe conditions, and meaningful engagement.

Complementing this view, Duffy et al. (2016) Psychology of Working Theory (PWT) emphasizes how career adaptability serves as a key mechanism in transforming structural barriers and personal resources into pathways toward decent work attainment. PWT highlights that adaptability activates psychological resources, such as self-regulation and proactive behaviors, to foster perceptions of future decent work, particularly in uncertain or resource-constrained contexts. By building on survival needs and promoting volitional control over career choices, adaptability strengthens individuals' beliefs in their ability to secure sustainable and fulfilling employment, thus linking adaptive capacities to positive future-oriented cognitions.

Empirical research offers growing support for this theorized link. For instance, a study focusing on college students from low-income families found that career adaptability directly and positively predicted their perceptions of future decent work (Wei et al., 2022). This finding suggests that adaptability resources empower individuals to envision and believe in the possibility of obtaining fulfilling and dignified work despite potential socioeconomic barriers. Similarly, in a sample of rural-oriented pre-service teachers in China, career adaptability positively predicted perceptions of future decent work, highlighting the role of adaptability in shaping positive work-related expectations and outcomes within educational career paths amid challenges like resource disparities and role transitions (Wen et al., 2024).

Hence, Hypothesis 4 (H4): career adaptability and perceived future decent work sequentially and positively mediate the relationship between career calling and academic resilience.

### 2.5 The moderating role of decent work

Decent work provides foundational resources and protections that enhance individuals' capacity to buffer psychological strain under pressure (Duffy et al., 2016), thereby functioning as a contextual amplifier that moderates the associations between other positive variables and academic resilience. The construct referenced here—present perceptions/experiences of decent work—is distinct from perceived future decent work discussed earlier. Whereas the latter concerns anticipated access and expectations regarding decent employment after graduation (i.e., the prospect of securing safe, fair, dignified, and sustainable work), and thus reflects a forward-looking judgment, the former focuses on the decency of one's current study/work context—including safety and health protections, fair compensation, reasonable hours and rest, social support and respect, and growth opportunities—and represents a subjective appraisal of the immediate environment. Building on Bronfenbrenner's (1979) ecological systems theory—which posits that the quality of the microsystem moderates links between individual attributes and adaptive outcomes—decent work emerges as a crucial environmental factor that helps individuals navigate career challenges while enabling the realization of personal and social value. For example, safe workplaces, as a core element of decent work, have demonstrated significant moderating effects on work-related wellbeing (Tebbe et al., 2019).

The core mechanism of this moderating role is that when individuals perceive their current study or work setting to embody features of decent work, such perceptions provide superior conditions and a stronger platform for other psychological resources and positive expectations to operate (Duffy et al., 2016), thereby magnifying and stabilizing their beneficial effects on academic resilience. Among doctoral students, decent work can serve as a resource enabler: higher levels of perceived decency signal greater economic security and social protection, which buffer anxiety and interference arising from unmet basic needs (Duffy et al., 2016). This shift allows students to redirect psychological energy from “survival worries” to “scholarly pursuits,” smoothing the translation of calling-driven motivation into resilient academic behavior. Decent work also reinforces value recognition and efficacy. In line with Career Construction Theory (Savickas, 2005), doctoral students' adaptive functioning represents psychosocial resources for meeting challenges; when present conditions convey respect and recognition of personal value (Duffy et al., 2016), these conditions bolster self-efficacy, strengthening students' conviction that their efforts will be met with appropriate rewards and respect. As a result, they deploy their resources more fully and confidently to overcome academic difficulties, thereby fortifying the pathway from career calling to academic resilience. In addition, current decent work operates as a “reality anchor” and validator of hope. Prior work suggests that perceived future decent work positively predicts resilience (Wen et al., 2024), and present decent work provides empirical grounding for such positive expectations (Wen et al., 2023). This consistency between present reality and future expectations consolidates hope (Feldman and Snyder, 2005), rendering the calling-inspired future more credible and compelling, and in turn strengthening the chain through which perceived future decent work promotes academic resilience.

Accordingly, the following hypotheses are proposed:

Hypothesis 5 (H5): Decent work positively moderates the relationship between career calling and academic resilience.

Hypothesis 6 (H6): Decent work positively moderates the relationship between career adaptability and academic resilience.

Hypothesis 7 (H7): Decent work positively moderates the relationship between perceived future decent work and academic resilience.

**Figure 1:**
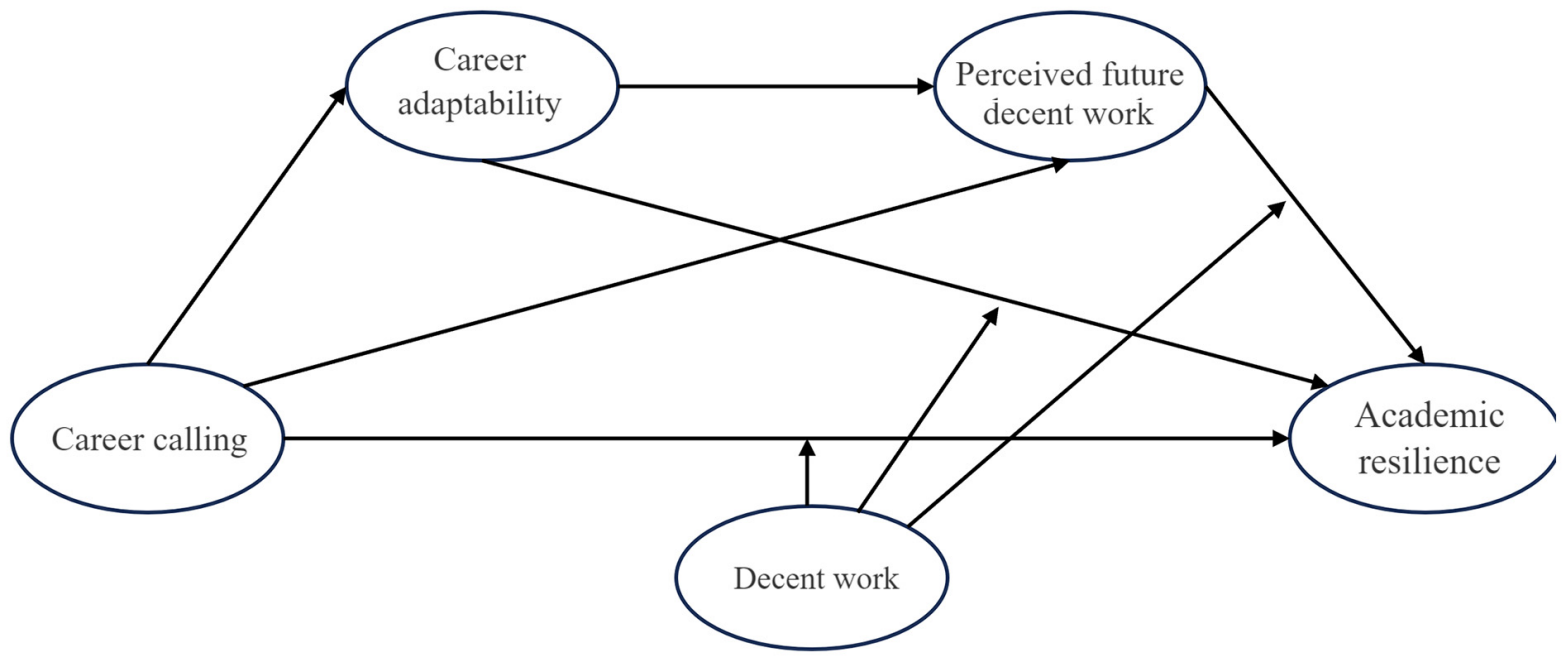
Theoretical model of this study.

Based on previous literature, this study proposes the theoretical model (Figure 1).

## 3 Materials and methods

### 3.1 Participants

This study adopted a cross-sectional survey design and recruited 898 doctoral candidates from multiple universities in China through convenience sampling between October 9, 2024, and January 5, 2025. To distribute the instruments and obtain responses, the following procedures were implemented, combining online and offline methods:

For online distribution, the Wenjuanxing short link/QR code was first shared within the doctoral community of the researcher's university, specifically in doctoral cohort WeChat groups (by year), lab/research-team chats, and methods/seminar groups. It was then broadened through WeChat/QQ friend lists and Moments and doctoral academic-exchange groups relevant to disciplines or research methods. Where required, permission from group administrators was sought, and group-based sharing was limited to academic or research-related groups. To widen reach, announcements were also posted on public social-media platforms (e.g., Zhihu, Xiaohongshu, Weibo). Two gentle reminders were issued around Day 7 and Day 14, and no direct or repeated one-to-one messages were sent to avoid pressure.

For offline distribution, paper questionnaires were handed out during university seminars, doctoral program meetings, and academic events (lectures, workshops, forums), with the same cover sheet and instructions. The online landing page presented an informed-consent statement (purpose, voluntariness, anonymity, confidentiality), and participation required checking a mandatory consent box before items appeared; paper respondents signed the same statement.

Electronic responses were captured automatically via Wenjuanxing, whereas paper forms were double-entered and reconciled. To safeguard data integrity, Wenjuanxing settings limited duplicate submissions (device/IP checks; no back-navigation resubmission), and no personal identifiers were collected during online distribution. Ex-ante/ex-post quality screens excluded cases with >20% missing data, inconsistent/patterned responding, or completion times < 5 min; no incentives were offered. After screening, 865 valid cases remained (96.3%).

Participants were stratified by academic year: 26% first-year, 25% second-year, 28% third-year, and 21% fourth-year or above. The age distribution was as follows: ≤ 25 years (*n* = 87, 10.1%), 26–27 years (*n* = 346, 40.0%), 28–29 years (*n* = 277, 32.0%), and ≥30 years (*n* = 155, 17.9%). In terms of gender, the sample comprised 447 males (51.7%) and 418 females (48.3%). Participants' universities were distributed across China's major regions: eastern (*n* = 536, 62.0%), central (*n* = 199, 23.0%), and western (*n* = 130, 15.0%). All participants were enrolled in academic/research-oriented doctoral programs (*n* = 865, 100%). No minors participated in the study.

### 3.2 Instruments

All scales used validated Chinese versions or were back-translated following standard translation procedures by two bilingual experts, with discrepancies resolved by consensus. A pilot with 60 doctoral students confirmed clarity and cultural fit (all α > 0.70). Confirmatory factor analyses in AMOS 26.0 verified construct validity. Model fit was judged using commonly referenced benchmarks (Hu and Bentler, 1999; Kline, 2018): χ^2^/df < 5 as acceptable (with < 3 indicating good fit and < 2 excellent), RMSEA < 0.08 as acceptable (with < 0.05 good), and incremental fit indices (NFI, IFI, TLI, CFI) > 0.90 as acceptable (with > 0.95 good). For multidimensional scales, hierarchical omega values above 0.50 were considered supportive of higher-order factor structures (Revelle and Zinbarg, 2009), justifying the use of total scores. Full results are reported in Table 1.

**Table 1:** Summary of CFA results and ω_h values.

**Scale**	**Model type**	**χ^2^/df**	**RMSEA**	**NFI**	**IFI**	**TLI**	**CFI**	**ω_h**
Career calling scale	Single-factor	2.647	0.044	0.987	0.992	0.990	0.992	N/A
Career adapt-abilities Scale–short form	First-order (Four-factor)	3.024	0.048	0.893	0.926	0.916	0.925	N/A
	Second-order	3.008	0.048	0.893	0.926	0.917	0.925	0.712
Perceived future decent work scale	First-order (Five-factor)	3.211	0.051	0.977	0.984	0.979	0.984	N/A
	Second-order	3.158	0.050	0.977	0.984	0.980	0.984	0.861
Academic resilience scale	First-order (Three-factor)	2.354	0.040	0.897	0.938	0.933	0.938	N/A
	Second-order	2.354	0.040	0.897	0.938	0.933	0.938	0.706
Decent work scale	First-order (Five-factor)	2.704	0.044	0.978	0.986	0.982	0.986	N/A
	Second-order	2.591	0.043	0.978	0.986	0.983	0.986	0.654

#### 3.2.1 Career calling scale

Developed by (Dobrow and Tosti-Kharas 2011), this scale includes 12 context-specific items validated across four student groups (music, management, business, and art). It was translated and back-translated following Brislin's procedure to ensure linguistic and conceptual equivalence in the Chinese doctoral context. In the present study, the items were adapted to measure calling toward an academic career. Using a 7-point Likert scale (1 = strongly disagree, 7 = strongly agree), higher scores indicate stronger vocational calling. Example items include: “I am passionate about pursuing an academic career” and “I would sacrifice everything to be an academic.” The Cronbach's alpha coefficient of the scale was 0.973.

#### 3.2.2 Career adapt-abilities scale–short form

Developed by Yu et al. (2019), this 12-item scale measures four dimensions: concern, control, curiosity, and confidence. The Chinese-validated version (Yu et al., 2019) was adopted with minor contextual adjustments for use in the present study. Using a 5-point Likert scale (1 = strongly disagree, 5 = strongly agree), higher scores reflect better career adaptability. Example items include: “Thinking about what my future will be like” (concern) and “Making decisions by myself” (control). The Cronbach's alpha coefficient in this study was 0.886.

#### 3.2.3 Perceived future decent work scale

Developed by Wei et al. (2022), this 15-item scale evaluates five dimensions: safe working conditions, access to healthcare, adequate compensation, free time and rest, and complementary values. This instrument was originally developed and validated in Chinese, requiring no translation. Using a 7-point Likert scale (1 = strongly disagree, 7 = strongly agree), higher scores indicate better perceived future decent work. Example items include: “I will feel emotionally safe interacting with people at my future work” (safe working conditions) and “I will have a good healthcare plan at future work” (access to healthcare). The Cronbach's alpha coefficient was 0.940.

#### 3.2.4 Academic resilience scale

Based on Cassidy (2016), this 30-item scale measures three dimensions: persistence, reflecting and adaptive help-seeking, and negative affect and emotional response. The English version was translated and back-translated for this study and checked for conceptual equivalence. Using a 5-point Likert scale (1 = strongly disagree, 5 = strongly agree), higher scores indicate a greater capacity for positive adaptation to academic adversity (some items are reverse-scored). Example items include: “I know I have the ability to complete this course successfully” (persistence) and “I have some doubts about my ability to do the work” (negative affect and emotional response; reverse-scored). The Cronbach's alpha coefficient was 0.940.

#### 3.2.5 Decent work scale

Developed by Duffy et al. (2017), this 15-item scale assesses five dimensions: physically and interpersonally safe working conditions, access to health care, adequate compensation, hours that allow for free time and rest, and organizational values that complement family and social values. The back-translated version was contextually adapted for use in the present study. Using a 7-point Likert scale (1 = strongly disagree, 7 = strongly agree), higher scores indicate better individual experiences of current engagement in decent work (some items are reverse-scored). Example items include: “I feel emotionally safe interacting with people at work” (physically and interpersonally safe working conditions) and “I have adequate health care coverage from my job” (access to health care). The Cronbach's alpha coefficient was 0.870.

### 3.3 Data analysis

This study employed SPSS 26.0 statistical software, along with Hayes‘ PROCESS macro for SPSS and Amos 26.0, to perform the following analyses: reliability and validity tests, descriptive statistics, correlation analysis, as well as serial mediation and moderation effect analyses. Specifically, reliability was assessed using Cronbach's alpha coefficients, while validity was evaluated through confirmatory factor analysis (CFA) in AMOS 26.0, with model fit was judged by indices such as RMSEA (< 0.08) and incremental fit indices (NFI, IFI, TLI, CFI > 0.90). Descriptive statistics and Pearson correlation analyses were conducted in SPSS 26.0 to examine means, standard deviations, and inter-variable relationships. For serial mediation, a structural equation model (SEM) was constructed in AMOS 26.0 to test path relationships, with model fit evaluated using the aforementioned indices. Mediation effects were further examined using the Bootstrap method in AMOS 26.0; all Bootstrap analyses in this study involved resampling 5,000 times at a 95% confidence level with bias-corrected confidence intervals, where indirect effects were deemed significant if the confidence interval excluded zero, and partial mediation was indicated if the direct effect's interval also excluded zero. For moderation and moderated mediation, Hayes' PROCESS macro (Model 89) was applied in SPSS 26.0, incorporating interaction terms to assess conditional effects at mean ± 1 SD levels of the moderator (decent work), with significance determined via the aforementioned Bootstrap confidence intervals.

## 4 Results

### 4.1 Common method bias and testing

Since the data were collected through self-reported surveys, this study conducted common method bias testing. We conducted Harman's single-factor test by performing an exploratory factor analysis without rotation on all measurement items. The results showed that the first unrotated factor accounted for 19% of the total variance, which is below the commonly accepted threshold of 40%. This indicates that there is no serious common method bias in the study data.

### 4.2 Descriptive statistics and correlation analysis

Table 2 reports the means, standard deviations, and correlation analysis results of the variables: (1) Career calling demonstrated significant positive correlations with academic resilience (*r* = 0.389, *p* < 0.01), career adaptability (*r* = 0.308, *p* < 0.01), and perceived future decent work (*r* = 0.346, *p* < 0.01). (2) Career adaptability demonstrated significant positive correlations with academic resilience (*r* = 0.421, *p* < 0.01) and perceived future decent work (*r* = 0.393, *p* < 0.01). (3)Perceived future decent work was significantly positively correlated with academic resilience (*r* = 0.428, *p* < 0.01). These correlation results provide preliminary verification of the aforementioned hypotheses and establish the foundation for subsequent hypothesis testing.

**Table 2:** Means, standard deviations, and correlations.

**Variable**	**M**	**SD**	**1**	**2**	**3**	**4**
1 Academic resilience	3.349	0.701	-			
2 Career calling	4.895	1.602	0.389^**^	-		
3 Career adaptability	3.486	0.675	0.421^**^	0.308^**^	-	
4 Future decent work perceptions	4.561	1.386	0.428^**^	0.346^**^	0.393^**^	-

^**^ *p* < 0.01.

### 4.3 Serial mediation analysis of career calling, career adaptability, perceived future decent work, and academic resilience

**Figure 2:**
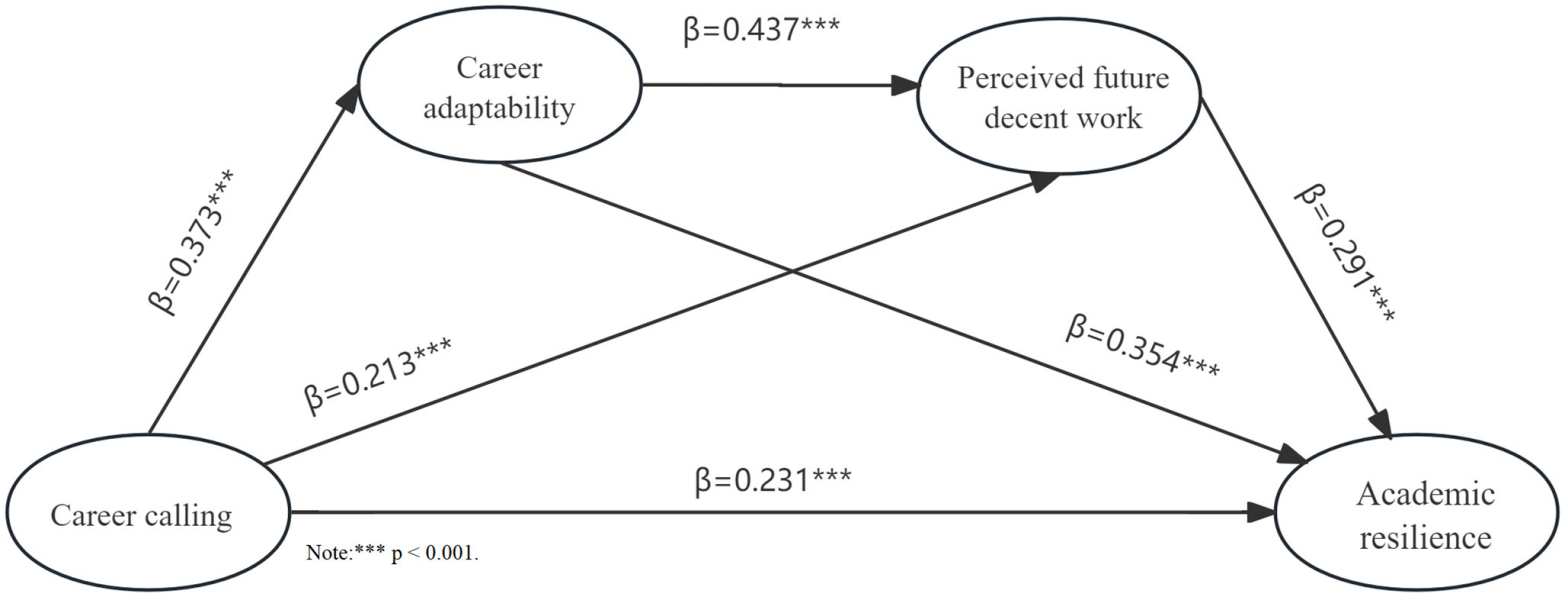
Results of the serial mediation model.

Using AMOS 26.0, we constructed and tested a full structural equation model with career calling as the independent variable, academic resilience as the dependent variable, and career adaptability and perceived future decent work as mediators. The serial mediation model demonstrated good fit: χ^2^/df = 2.744 (below 3); CFI = 0.970 (above 0.90); TLI = 0.961 (above 0.90); IFI = 0.970 (above 0.90); RMSEA = 0.045 (below 0.08). All indices met the recommended thresholds, indicating satisfactory model fit for examining the path relationships among variables. Given the satisfactory model fit, we proceeded to examine the direct and indirect effects among the variables, as illustrated in Figure 2 (detailed coefficients are presented in Sections 4.3.1–4.3.4).

Subsequently, this study employed the Bootstrap method in AMOS 26.0 to test the mediation effects, with the sample size set to 5,000 (typically requiringover 1,000) and the confidence interval level set to 95%, using bias-corrected confidence intervals to observe their upper and lower bounds. When the bias-corrected confidence interval of the indirect effect does not include 0, it indicates the presence of a mediation effect. For the direct effect's bias-corrected confidence interval: if it does not include 0, it signifies that the direct effect is also significant, indicating partial mediation; if it includes 0, it indicates complete mediation. The test results are shown in Table 3.

**Table 3:** Results of serial mediation effect analysis.

**Path**	**Effect value**	**95%Confidence interval**	
		**Boot LLCI**	**Boot ULCI**
Direct effect	0.231	0.127	0.297
Career calling → career adaptability → academic resilience	0.132	0.083	0.196
Career calling → future decent work perceptions → academic resilience	0.062	0.031	0.107
Career calling → career adaptability → future decent work perceptions → academic Resilience	0.047	0.029	0.074

#### 4.3.1 Analysis of the path “career calling → academic resilience”

The AMOS 26.0 analysis results shown in Figure 2 indicate that the standardized path coefficient from career calling to academic resilience was 0.231 (*p* < 0.001), demonstrating a significant positive effect. According to Table 3, the effect value for the “career calling → academic resilience” path was 0.231, with a 95% confidence interval of (0.127, 0.297) that does not include zero. This confirms that career calling has a positive impact on academic resilience. Both the structural equation modeling and bootstrap results consistently show that career calling has a significant effect on academic resilience, thus supporting Hypothesis H1.

#### 4.3.2 Analysis of the path “career calling → career adaptability → academic resilience”

The AMOS 26.0 results presented in Figure 2 show that the path coefficient from career calling to career adaptability was 0.373 (*p* < 0.001), and from career adaptability to academic resilience was 0.354 (*p* < 0.001), both reaching statistical significance. Table 3 shows that the effect value for the “career calling → career adaptability → academic resilience” path was 0.132, with a 95% confidence interval of (0.083, 0.196) that excludes zero. Both the structural equation modeling and bootstrap results confirm the significant mediating role of career adaptability, thereby supporting Hypothesis H2.

#### 4.3.3 Analysis of the path “career calling → perceived future decent work → academic resilience”

As shown in the test results of AMOS 26.0 in Figure 2, the path coefficient of career calling on perceived future decent work is 0.213 (*p* < 0.001), and the path coefficient of perceived future decent work on academic resilience is 0.291 (*p* < 0.001). Meanwhile, according to Table 3, the effect value of the path “Career calling → Perceived future decent work → Academic resilience” is 0.062, and the 95% confidence interval is (0.031, 0.107), which does not contain 0. Both the results of the structural equation model and the Bootstrap method show that the mediating effect of perceived future decent work is significant, and Hypothesis H3 is supported.

#### 4.3.4 Analysis of the path “career calling → career adaptability → perceived future decent work → academic resilience”

According to the test results of AMOS 26.0 in Figure 2, the path coefficient of career adaptability on perceived future decent work is 0.437 (*p* < 0.001). Meanwhile, combined with the Bootstrap results in Table 3, the serial mediating effect value of the path “Career calling → Career adaptability → Perceived future decent work → Academic resilience” reaches 0.047, and the 95% confidence interval is (0.029, 0.074), which does not contain 0. Based on the previous analysis, the serial mediating effect of career adaptability and perceived future decent work is significant, and Hypothesis H4 is supported.

### 4.4 Test of the moderating effect of decent work

This study used Model 89 of the SPSS macro PROCESS to examine the moderating effect on this serial mediation. As shown in Table 4, after incorporating decent work perception into the serial mediation model, the overall model with academic resilience as the outcome variable yielded an R^2^ = 0.318, indicating that the model explains 31.8% of the variance. The F-value was 0.318 (*p* < 0.001), reaching statistical significance.

**Table 4:** Results of the moderating effect test of decent work.

**Regression equation**		**Overall model fit indices**			**Coefficient significance**	
**Outcome variable**	**Independent variables**	**R**	**R** ^2^	**F(df)**	β	**t**
Academic resilience		0.564	0.318	57.141		
	Career calling	-	-	-	0.211	6.502^***^
	Career adaptability	-	-	-	0.235	7.126^***^
	Future decent work perceptions	-	-	-	0.240	7.212^***^
	Decent work	-	-	-	0.133	3.423^***^
	Decent work × career calling	-	-	-	0.068	2.091^*^
	Decent work × career adaptability	-	-	-	0.072	2.194^*^
	Decent work × future decent work perceptions	-	-	-	0.067	2.139^*^

^*^ *p* < 0.05;^***^ *p* < 0.001.

#### 4.4.1 The moderating effect of decent work on the relationship between career calling and academic resilience

The product term of career calling and decent work had a significant positive effect on academic resilience (β = 0.068, *p* < 0.05), indicating that decent work positively moderates the relationship between career calling and academic resilience. Thus, Hypothesis H5 was supported. The effect values of career calling on academic resilience at different levels of decent work and their 95% Bootstrap confidence intervals are presented in Table 5.

**Table 5:** The moderating effect of decent work on the relationship between career calling and academic resilience.

**Decent work**	**Effect value**	**Boot SE**	**Boot LLCI**	**Boot ULCI**
M-1SD	0.063	0.048	0.022	0.104
M	0.092	0.033	0.064	0.120
M+1SD	0.122	0.044	0.084	0.160

**Figure 3:**
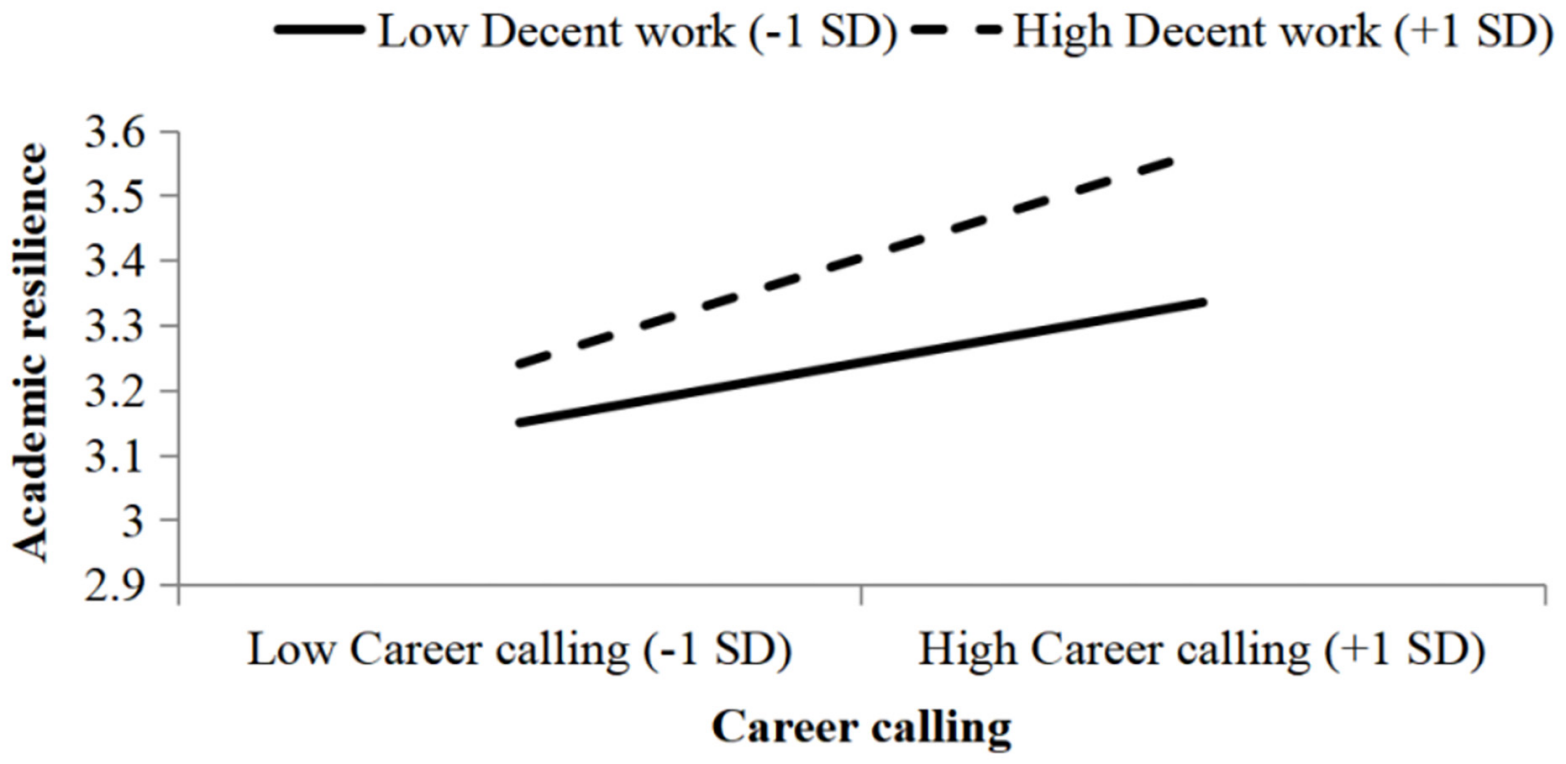
The moderating effect of decent work on the relationship between career calling and academic resilience.

To better illustrate the moderating effect of decent work, Figure 3 plots the moderating effect using the mean of decent work perception plus/minus one standard deviation as grouping variables.

As shown in Figure 3, the relationship between career calling and academic resilience varied under different levels of decent work perception. When decent work perception was high, the effect of career calling on academic resilience was stronger; when decent work perception was low, the effect was weaker. This confirms that decent work indeed serves as a positive moderator between career calling and academic resilience. Therefore, Hypothesis H5 was further supported.

#### 4.4.2 The moderating effect of decent work on the relationship between career adaptability and academic resilience

The interaction term between career adaptability and decent work demonstrated a significant positive effect on academic resilience (β =0.072, *p* < 0.01), indicating that decent work plays a positive moderating role between career adaptability and academic resilience, supporting Hypothesis H6. The effect sizes of career adaptability on academic resilience at different levels of decent work and their 95% Bootstrap confidence intervals are shown in Table 6.

**Table 6:** The moderating effect of decent work on the relationship between career adaptability and academic resilience.

**Decent work**	**Effect value**	**Boot SE**	**Boot LLCI**	**Boot ULCI**
M-1SD	0.169	0.048	0.075	0.263
M	0.244	0.034	0.177	0.311
M+1SD	0.319	0.049	0.223	0.415

**Figure 4:**
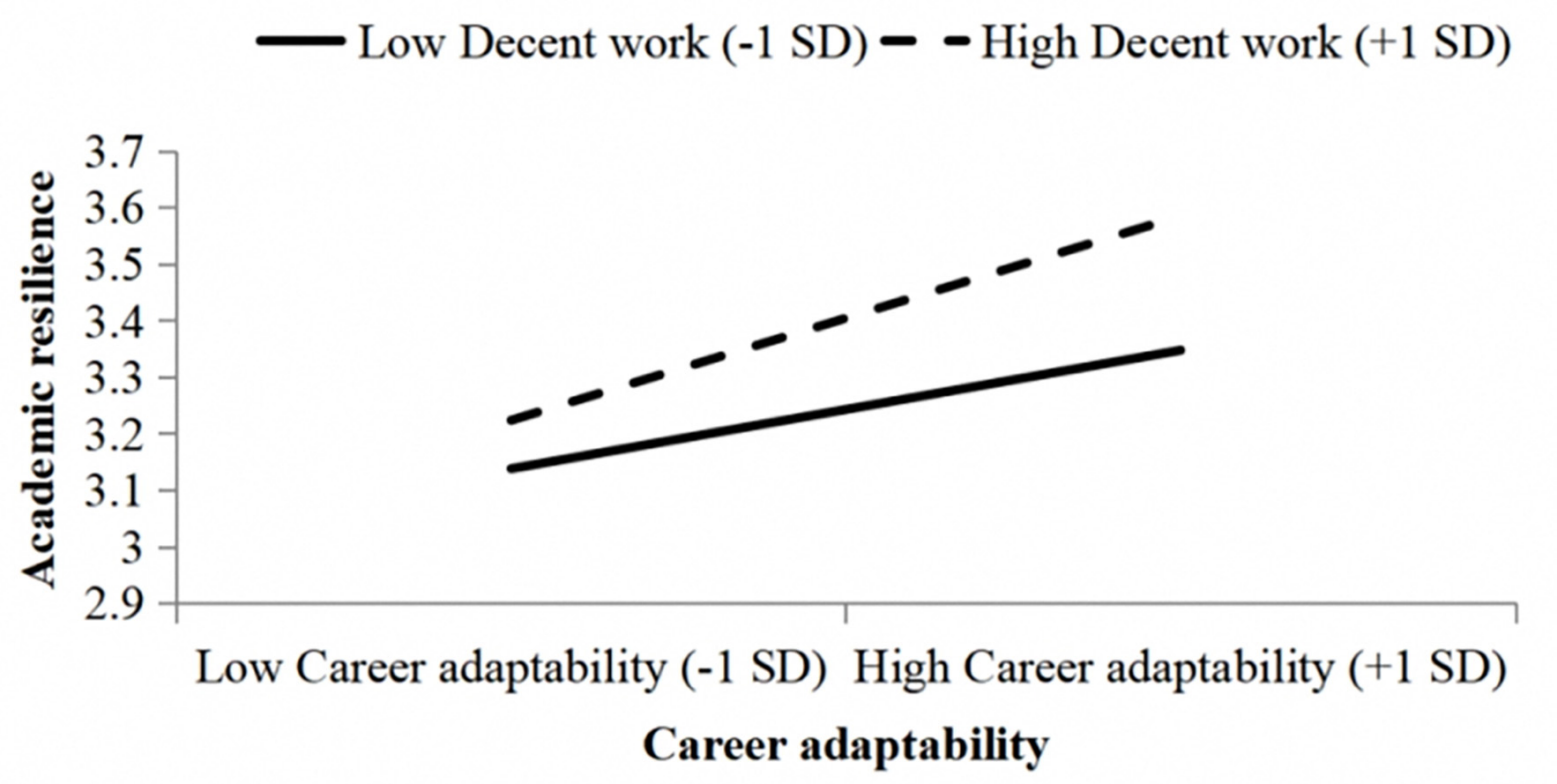
The moderating effect of decent work on the relationship between career adaptability and academic resilience.

To better demonstrate the moderating effect of decent work, as shown in Figure 4, the moderating effect was plotted using the mean of decent work level plus and minus one standard deviation as grouping variables.

As shown in Figure 4: the differences in the relationship between career adaptability and academic resilience under different levels of decent work. When the level of decent work was higher, the effect of the former on the latter was stronger; when the level of decent work was lower, the effect was weaker, confirming that decent work indeed plays a positive moderating role between career adaptability and academic resilience. Therefore, Hypothesis H6 was further supported.

#### 4.4.3 The moderating effect of decent work on the relationship between perceived future decent work and academic resilience

The interaction between perceived future decent work and decent work was significantly and positively associated with academic resilience (β = 0.067, *p* < 0.05), indicating that decent work moderates the association between perceived future decent work and academic resilience. Hypothesis H7 was supported. To better demonstrate the moderating effect of decent work, Table 7 presents the effect sizes and 95% Bootstrap confidence intervals of perceived future decent work on academic resilience at different levels of decent work.

**Table 7:** The moderating effect of decent work on the relationship between perceived future decent work and academic resilience.

**Decent work**	**Effect value**	**Boot SE**	**Boot LLCI**	**Boot ULCI**
M-1SD	0.088	0.023	0.043	0.132
M	0.121	0.017	0.088	0.154
M+1SD	0.155	0.023	0.109	0.201

**Figure 5:**
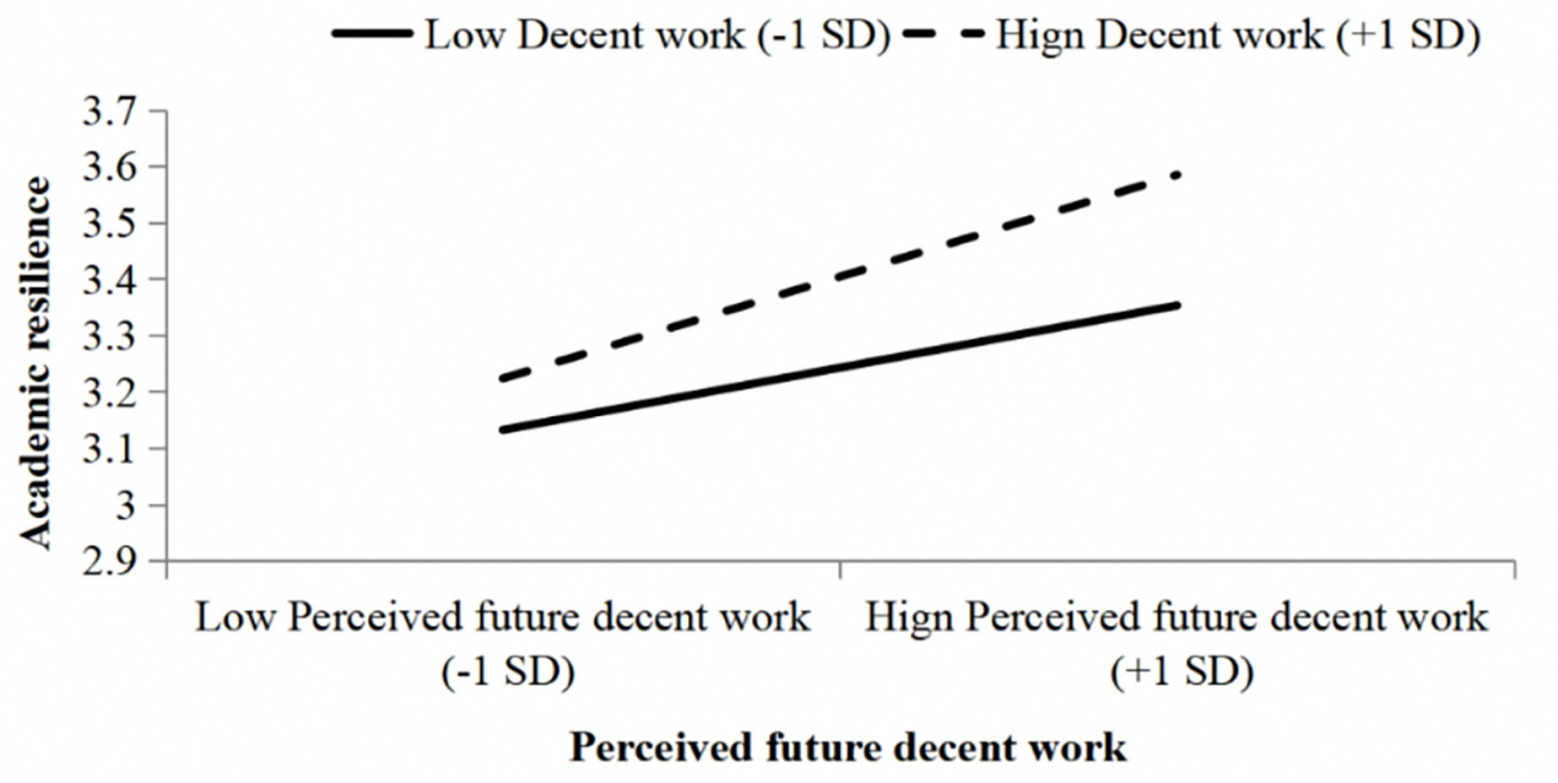
The moderating effect of decent work on the relationship between perceived decent work and academic resilience.

As illustrated in Figure 5, the moderating effect was plotted by categorizing the data at one standard deviation above and below the mean of decent work. As shown in Figure 5: the differences in the relationship between perceived future decent work and academic resilience under different levels of decent work. When the level of decent work was higher, the effect of perceived future decent work on academic resilience was stronger; when the level of decent work was lower, the effect was weaker, confirming that decent work indeed plays a positive moderating role between perceived future decent work and academic resilience. Therefore, Hypothesis H7 was further supported.

#### 4.4.4 Conditional indirect effects of career calling on academic resilience at three levels of decent work

We probed the conditional indirect effects for all hypothesized indirect paths (H2-H4). As shown in Table 8, the indirect effects were positive and statistically significant at low (−1 SD), mean, and high (+1 SD) levels of present decent work for each path. Although point estimates were numerically larger at higher levels of present decent work, the indices of moderated mediation had 95% CIs that included zero, indicating that differences in the magnitude of the indirect effects across levels of present decent work were not statistically reliable. Taken together, these exploratory results suggest that the mediation pathways operate consistently across levels of present decent work, while any amplification at higher levels is modest and non-significant.

**Table 8:** Conditional indirect effects of career calling on academic resilience at three levels of decent work.

**Indirect path**	**Decent work level**	**Effect**	**BootSE**	**95% BootLLCI**	**95% BootULCI**
Career calling → career adaptability → academic resilience	Low (M – 1 SD)	0.0223	0.0076	0.0091	0.0386
	Mean (M)	0.0318	0.0065	0.0199	0.0454
	High (M + 1 SD)	0.0413	0.0088	0.0250	0.0595
Index of Moderated Mediation	—	0.0097	0.0051	−0.0009	0.0194
Career calling → perceived future decent work → academic resilience	Low (M – 1 SD)	0.0184	0.0076	0.0061	0.0359
	Mean (M)	0.0257	0.0058	0.0157	0.0387
	High (M + 1 SD)	0.0329	0.0075	0.0194	0.0485
Index of moderated mediation (IMM)	—	0.0074	0.0049	−0.0030	0.0162
Career calling → career adaptability → perceived future decent work → academic resilience	Low (M – 1 SD)	0.0072	0.0029	0.0025	0.0139
	Mean (M)	0.0101	0.0023	0.0060	0.0153
	High (M + 1 SD)	0.0129	0.0032	0.0075	0.0198
Index of moderated mediation (IMM)	—	0.0029	0.0020	−0.0012	0.0067

## 5 Discussion

This study systematically reveals the mechanism through which career calling influences doctoral students' academic resilience by constructing a moderated serial mediation model.

### 5.1 The relationship between career calling and academic resilience

The findings demonstrate that career calling is significantly and positively associated with doctoral students' academic resilience. This result validates Dalla Rosa, Galliani and Vianello's (2014) proposition that career calling serves as a core psychological resource. From the perspective of Career Construction Theory, career calling can be situated within a person's life themes and narrative identity, which organize how individuals interpret and respond to developmental tasks, transitions, and traumas in their career (Savickas, 2005). Calling supplies a narrative scaffold that helps doctoral students implement their self-concept in academic work. It clarifies who they are becoming (vocational self), why the work matters (life-theme meaning), and how they should respond to recurrent adaptation episodes such as proposal setbacks, manuscript rejections, and null results (Price, 2023).

Three proximal pathways follow. First, narrative coherence reduces role confusion and sustains hierarchies of goals, supporting persistence amid ambiguity typical of long-cycle research (Savickas, 2005; McAdams and McLean, 2013). Second, narrative purpose strengthens self-regulatory routines—prioritizing core tasks, strategic help-seeking, and deliberate practice—so stressors are framed as story-consistent challenges rather than threats, facilitating cognitive reappraisal and continued effort (Dik and Duffy, 2009; Savickas, 2005). Third, narrative continuity over time enables students to integrate setbacks into a broader, meaningful life story, a process linked to better emotional recovery and wellbeing, which undergirds resilient academic engagement (Adler et al., 2016; McAdams and McLean, 2013; Price, 2023).

In sum, CCT suggests that calling is linked to academic resilience not only by enhancing motivation but also by giving adversity a place in one's career narrative. This narrative framing helps maintain engagement when external rewards are delayed or uncertain (Savickas, 2005).

### 5.2 The chain mediating effects of career adaptability and perceived future decent work

The study indicates that career calling is related to academic resilience through a serial process involving career adaptability and perceived future decent work.

First, career calling enhances academic resilience indirectly through career adaptability. Framed by Career Construction Theory (CCT), calling offers a coherent sense of “who I am becoming” and “why this work matters.” This clarity motivates doctoral students to mobilize adaptive resources and translate abstract vocational aspirations into concrete behaviors. According to CCT, doctoral students' career adaptability comprises four capacities: career concern (sustained responsibility for long-term academic goals), career control (agentic adjustment of research direction), career curiosity (openness to explore new fields or methods), and career confidence (belief in overcoming research setbacks; Savickas, 2005). This means that students with a strong sense of calling tend to set and protect long-term goals (concern), recalibrate strategies when facing obstacles (control), pursue interdisciplinary or methodological innovations (curiosity), and persist through failed experiments or critical feedback (confidence). Because career calling anchors academic work in enduring purpose and identity, doctoral students are more likely to view setbacks as role-consistent challenges. Such appraisals promote cognitive flexibility and reframing, reduce stress reactivity, and conserve personal resources, thereby strengthening academic resilience (Dik and Duffy, 2009; Price, 2023).

Second, career calling promotes academic resilience indirectly via perceived future decent work. CCT emphasizes future design and narrative projection—individuals actively imagine and script plausible work lives and then move toward those designs (Savickas, 2005). In this light, perceived future decent work operates as a future-oriented appraisal embedded in the student's career narrative: students who experience a stronger calling may construct more coherent and optimistic stories about securing fair, meaningful, and sustainable work conditions after graduation, and such expectations align the present effort with a valued future (Wei et al., 2022; Duffy et al., 2016). This prospective meaning-making helps students interpret near-term academic stressors as steps along a meaningful trajectory. This process aligns with the positive association observed between perceived future decent work and academic resilience.

Third, career calling influences academic resilience through the serial mediation of career adaptability followed by perceived future decent work. Career construction theory integrates these elements by emphasizing how adaptability in the present builds a coherent career narrative that extends into future expectations. In this path—illustratively in doctoral training contexts—students leverage adaptability to achieve tangible milestones (e.g., publishing papers or securing collaborations), which in turn strengthen perceptions of future decent work, such as stable academic positions or impactful contributions (Wen et al., 2024). This cumulative process creates a resilient ecosystem, where immediate adaptive behaviors reinforce long-term optimism, enabling students to reinterpret setbacks as steps toward a constructed career identity that sustains motivation and perseverance (Duffy et al., 2016).

### 5.3 The moderating effect of decent work

This study demonstrates that decent work experiences significantly strengthen the positive associations between career calling, career adaptability, perceived future decent work, and academic resilience. In line with recent research, meeting safety and material needs is foundational for engagement and persistence (Loofbourrow, 2023; Repella et al., 2024; McKay et al., 2025), moreover contextual supports—beyond individual traits—are important for adaptive functioning (Awais et al., 2024).

Extending this to doctoral education, microsystem quality—supervision climate and peer support—proves pivotal for doctoral students' day-to-day adaptation and wellbeing; in addition, supportive working conditions (e.g., scholarships, facilities, and training) at the exosystem level further enable positive adjustment (Newlands et al., 2025; Xu W., et al., 2024). Together, these studies underscore decent work as a proximal contextual resource shaping how individual strengths translate into resilience. Drawing on Bronfenbrenner's (1979) ecological systems theory, these moderating effects can be understood through the interplay of microsystem elements in shaping adaptive outcomes. In this study, decent work functions as a high-quality microsystem that moderates the relationships by facilitating a co-evolutionary dynamic between environmental provisions (e.g., dignity-preserving policies and supportive academic structures) and individual agency (e.g., intrinsic motivation from career calling). Specifically, when doctoral students perceive their current work as decent, it enhances the translation of career calling into resilient behaviors by providing a stable foundation for meaning-making. It also bolsters career adaptability by enabling effective strategy implementation amid challenges. In addition, it reinforces perceived future decent work by creating a positive feedback loop in which present support builds confidence in long-term vocational fulfillment. From the vantage point of Career Construction Theory (Savickas, 2005), decent work in the current academic context functions as a narrative-supportive context that sustains the student's ongoing project of implementing the self in work. Conversely, when present conditions undermine dignity or safety, the holding power of the narrative weakens, and the same personal dispositions or expectations yield smaller resilience advantages. Complementing this perspective, Maslow's (1954) hierarchy of needs explains how decent work facilitates resource reallocation. By satisfying foundational needs, it frees cognitive and emotional resources that sustain calling, adaptability, and future work expectations as motivational drivers of resilience.

## 6 Implications in practice

Grounded in this study's findings, the first implication focuses on strengthening calling-oriented education. The significant association between career calling and academic resilience suggests that calling should not be treated as an implicit or accidental outcome of doctoral study. Instead, universities may provide structured opportunities that help students explore the meaning and purpose of their academic work. Examples include reflective supervision, brief guided reflective writing about academic purpose, and narrative-based mentoring conversations. These activities can support students in understanding how their research aligns with personal values and long-term academic goals. When meaning becomes clearer, academic challenges—such as delayed progress or manuscript rejection—may be seen as normal and meaningful parts of growth, rather than signs of failure. In this way, calling-oriented practices can help build more stable motivation and support resilient academic engagement.

Second, given that career adaptability emerged as a significant mediator between career calling and academic resilience, doctoral programs may benefit from deliberately cultivating adaptability-related competencies. Training may focus on strengthening future-oriented planning, autonomous decision-making, exploratory behaviors, and confidence in navigating uncertainty. Such competencies can be developed through targeted workshops, peer-led motivational interviewing, or interdisciplinary collaboration opportunities. Over time, these practices may help students build the ability to respond flexibly and constructively to academic demands.

A third implication is linked to perceived future decent work. Students' expectations regarding fair, meaningful, and sustainable employment appear to influence their willingness to persist when facing academic difficulty. Transparent communication about both academic and non-academic pathways, labor conditions, and long-term career prospects may reduce uncertainty and reinforce future-oriented motivation.

Finally, the moderating effect of present decent work signals the importance of supportive institutional environments. When workload, supervision quality, compensation, and psychological safety meet reasonable standards, the positive effects of calling and adaptability are amplified. Therefore, resilience-building efforts should combine individual capacity development with structural improvements rather than relying on personal resources alone.

## 7 Limitations and future directions

First, the composition and recruitment of the sample may constrain external validity. Participants were recruited through convenience sampling from several Chinese universities rather than probability-based procedures. Because the sample consisted exclusively of research-oriented (academic) doctoral candidates, the findings may not generalize to professional or clinical doctorates, other educational stages, or non-Chinese contexts. Future research should consider stratified or probability sampling, targeted oversampling of underrepresented groups, and, where appropriate, post-stratification weighting to enhance representativeness.

Second, the study relied on self-report, single-source measures collected at one time point. Such designs are susceptible to common-method and shared-source bias (e.g., social desirability, transient mood, reference-group effects). Future work could mitigate these risks by incorporating multi-source data (e.g., advisor or peer ratings), behavioral or archival indicators (e.g., milestone timelines, submission/revision records), and procedural remedies such as temporal separation or marker variables.

Third, this study used a cross-sectional design to examine the influence of career calling on doctoral students' academic resilience. Although the research was guided by established theoretical and empirical frameworks and applied validated measurement instruments and appropriate analytical procedures, the inherent limitations of cross-sectional data remain. Specifically, such designs reveal associations rather than causal relationships. To clarify the dynamic processes through which career calling affects academic resilience, future research should adopt longitudinal designs that follow the same individuals over multiple time points, enabling stronger inferences about causal pathways and developmental trajectories.

Fourth, cultural determinants were not integrated as focal variables within the current analytical framework. Subsequent studies could enrich the model by incorporating cultural dimensions—such as organizational spiritual culture, institutional culture, and material culture—within academic settings. Moreover, while this study focused on doctoral students, future research could extend to other professional domains or educational stages. Comparative work across occupations and developmental stages would help assess the generalizability of career calling's influence on academic resilience and illuminate its mechanisms across diverse occupational and cultural contexts, offering both theoretical insights and practical guidance for broader populations.

## 8 Conclusion

This study elucidates the mechanisms through which career calling influences doctoral students' academic resilience, highlighting the serial mediating roles of career adaptability and perceived future decent work, as well as the moderating effect of decent work. Central to these findings is the dual effects of endogenous and exogenous motivation in fostering resilience. Endogenous motivation, primarily embodied in career calling and career adaptability, originates from within the individual: career calling provides intrinsic purpose, passion, and a sense of meaning that drives doctoral students to persevere through academic adversities, while career adaptability equips them with internal resources for flexible goal adjustment, curiosity-driven exploration, and confident problem-solving, serving as a mediator in the process.

Complementing this, exogenous motivation arises from external environmental factors and instrumental considerations, encompassing decent work as a moderator and perceived future decent work as a mediator. Decent work supplies supportive contexts such as fair compensation, safe conditions, and balanced workloads, while perceived future decent work represents instrumental value and rationality—anticipating external rewards and conditions that motivate practical goal pursuit and buffer stressors. Together, these exogenous elements amplify endogenous motivations by providing tangible incentives and validations, facilitating the conversion of personal strengths into resilient behaviors.

By leveraging these dual motivations—endogenous for intrinsic drive and exogenous for instrumental reinforcement—academic aspirations are bolstered, competencies enhanced, and passion nurtured, ultimately cultivating robust academic resilience among doctoral candidates. These insights underscore the need for integrated interventions that nurture both internal psychological resources and external systemic supports, offering a holistic framework for doctoral education reform.

## Data Availability

The raw data supporting the conclusions of this article will be made available by the authors, without undue reservation.
